# Neuroinflammation and Neurodegeneration: Pinpointing Pathological and Pharmacological Targets

**DOI:** 10.1155/2015/487241

**Published:** 2015-07-30

**Authors:** Antonio Carlos Pinheiro de Oliveira, Eduardo Candelario-Jalil, Bernd L. Fiebich, Magda da Silva Santos, András Palotás, Helton José dos Reis

**Affiliations:** ^1^Department of Pharmacology, Federal University of State Minas Gerais, Avenida Antonio Carlos 6627, 31270-901 Belo Horizonte, MG, Brazil; ^2^Department of Neuroscience, University of Florida, Gainesville, FL 32610, USA; ^3^Department of Psychiatry, University of Freiburg Medical School, Hauptstrasse 5, 79104 Freiburg, Germany; ^4^Department of Psychiatry, University of California San Francisco, School of Medicine, San Francisco, CA 94143-0984, USA; ^5^Asklepios-Med, Kossuth Lajos Sugárút 23, 6722 Szeged, Hungary

For many years, the brain had been regarded as an immune-privileged organ because of an old tenet which stated that no classical immune activation or inflammation could take place intrathecally. However, this theory has quickly changed with the advent of several studies demonstrating that the central nervous system (CNS) is in fact immunologically specialized [1, 2].

Neuroinflammation has been viewed as a double-edged sword: it not only is essential for the recovery from a number of conditions, but also may play detrimental roles in neurodegenerative processes. In such disorders inflammation can be set off by versatile triggers: protein aggregates, mediators released from injured neurons, accumulation of abnormally modified cellular components, and suppression of mechanisms that would normally control inflammatory processes, just to mention a few [3].

Given the increased life-expectancy, the incidence of neurodegenerative diseases is steadily rising. In light of this, research into this large segment of neuropsychiatry is a top priority around the globe, and one of the main areas of focus is to understand neuroinflammation that underlies, at least in part, the most common degenerative conditions of the brain: Alzheimer's dementia, Parkinson's disease, amyotrophic lateral sclerosis, Huntington's chorea, and many others [4–6]. By addressing intrathecal inflammation, some of these disorders could be prevented or even successfully treated.

This special issue compiles original articles and reviews dissecting various pharmacological targets of inflammation that may serve as a springboard for opening innovative therapeutic avenues and could be germane to advanced research in neurodegenerative disorders.

*Antonio Carlos Pinheiro de Oliveira* *Eduardo Candelario-Jalil* *Bernd L. Fiebich* *Magda da Silva Santos* *András Palotás* *Helton José dos Reis*
